# S137. DO HALLUCINATIONS PREDICT THE TRANSITION FROM SUICIDAL THOUGHTS TO ATTEMPTS? RESULTS FROM AN AUSTRALIAN LONGITUDINAL COHORT STUDY

**DOI:** 10.1093/schbul/sby018.924

**Published:** 2018-04-01

**Authors:** Emily Hielscher, Jordan DeVylder, Melissa Connell, Penelope Hasking, Graham Martin, James Scott

**Affiliations:** 1 The University of Queensland; 2 Fordham University; 3 Curtin University

## Abstract

**Background:**

Although suicidal ideation is a well-documented risk factor for suicidal behaviour, the majority of those with suicidal thoughts do not go on to make an attempt. Therefore, it is important to improve prediction of which individuals are more likely to act on their suicidal thoughts, as highlighted in Klonsky and May’s (2015) ideation-to-action framework. Auditory hallucinations (AH) and psychological distress (PD) are strongly associated with both suicidal thoughts and behaviour, but their role in the ideation-to-attempt transition has not been investigated in a longitudinal dataset.

**Methods:**

Participants were from an Australian longitudinal cohort of 1793 adolescents (12–17 years). Suicidal thoughts and behaviours were measured using the Self-Harm Behaviour Questionnaire. The Diagnostic Interview Schedule for Children was used to assess AH. PD was categorised using the General Health Questionnaire (GHQ) clinical cut-off. Those reporting suicidal ideation were stratified into four groups: (i) Those who did not have PD or AH (reference group), (ii) AH only, (iii) PD only, and (iv) PD and AH. Using logistic regression, we examined associations between baseline suicidal ideation, and incident suicide attempts during the 12-month follow-up, stratified by the four comparison groups. All analyses were adjusted for age and sex.

**Results:**

AH were strongly and independently associated with baseline suicidal ideation (OR=3.84; 95%CI=2.46–6.02) and suicide attempts in the following 12 months (OR=3.21; 95%CI=1.18–8.76). Among adolescents with baseline suicidal ideation (n=235; 13.1%), 14 or 6.0% attempted suicide at follow-up. Those with AH only were not at significantly increased risk of transition from suicidal thoughts to attempts (OR=2.97; 95%CI=0.26–34.59). Similarly, adolescents with PD only did not have a significant increase in transition from ideation to attempts (OR=4.48; 95%CI=0.91–22.14). Adolescents who had both PD and AH had an eight-fold increased risk (OR=8.42; 95%CI=1.46–48.67) of acting on their suicidal thoughts.

**Discussion:**

Adolescents with both PD and AH had the greatest likelihood of acting on their suicidal thoughts. AH alone did not significantly predict the transition from suicidal thoughts to attempts despite high odds ratios, possibly due to the low prevalence of suicide attempts among ideators and consequently limited statistical power. Future studies examining for negative and distressing content of hallucinations may assist in explaining their role in the ideation-to-attempt transition. Screening adolescents who are distressed and have hallucinations may assist with predicting those at greatest risk of future suicide attempts.

